# Association of a variant in the gene encoding for ERV1/ChemR23 with reduced inflammation in visceral adipose tissue from morbidly obese individuals

**DOI:** 10.1038/s41598-017-15951-z

**Published:** 2017-11-16

**Authors:** Cristina López-Vicario, Bibiana Rius, José Alcaraz-Quiles, Ana González-Périz, Ana Isabel Martínez-Puchol, Mireia Casulleras, Marta Duran-Güell, Ainitze Ibarzabal, Ricard Corcelles, Andrés Laguna-Fernández, Magnus Back, Esther Titos, Joan Clària

**Affiliations:** 1 Department of Biochemistry and Molecular Genetics, Hospital Clínic, IDIBAPS, Barcelona, Spain; 2 Department of Gastrointestinal Surgery, Hospital Clínic, IDIBAPS, Barcelona, Spain; 3 CIBERehd, University of Barcelona, Barcelona, Spain; 4 Department of Biomedical Sciences, University of Barcelona, Barcelona, Spain; 5 Centre for Molecular Medicine, Department of Medicine, Karolinska Institutet and Department of Cardiology, Karolinska University Hospital, Stockholm, Sweden

## Abstract

Obesity comorbidities are closely associated with chronic low-grade adipose tissue inflammation. A number of SNPs associated with inflammation has been identified, underscoring the impact of genetic determinants on this process. Here, we screened SNPs in genes with pro-inflammatory (IL-1β, IL-6, STAT3 and JAK2), anti-inflammatory (IL-10 and SOCS3) and pro-resolving (ERV1/ChemR23) properties in 101 obese and 99 non-obese individuals. Among the SNPs analyzed, we identified that individuals carrying a C allele in the rs1878022 polymorphism of the ERV1/ChemR23 gene, which encodes for the receptor of the pro-resolving mediator RvE1, had increased ERV1/ChemR23 protein expression and reduced levels of the inflammatory cytokine IL-6 in adipose tissue. Moreover, patients carrying the C allele in homozygosity had lower plasma levels of IL-6, IFN-α2, IL-15, IL-1ra, IL-10, GM-CSF, G-CSF and VEGF and enhanced leukocyte responsiveness to RvE1. C-carriers also exhibited decreased TAG to HDL ratio, a surrogate marker of insulin resistance and a predictor of incident fatty liver. Finally, we confirmed *in vivo* that the ERV1/ChemR23 receptor regulates systemic and tissue inflammation since mice lacking ERV1/ChemR23 expression showed increased IL-6 levels in adipose tissue and peritoneal macrophages. Together, our study identified an ERV1/ChemR23 variant that protects patients with obesity from excessive inflammatory burden.

## Introduction

Obesity is defined as increased adipose tissue mass and is the net result of an imbalance between energy intake and energy expenditure. Obesity is a highly prevalent and rapidly evolving health concern, and in recent years, has acquired the characteristics of a global pandemic disease^1^. The health impact of this pandemic is not only the burden of excessive weight but also the predisposition to frequently present comorbidities such as insulin resistance and type 2 diabetes, dyslipidemia, non-alcoholic fatty liver disease, hypertension and cardiovascular disease^2^.

Recent studies have confirmed the positive association between comorbidities and inflammation in obesity. In this regard, insulin resistance and non-alcoholic fatty liver disease in obese individuals have been linked to a chronic state of low-grade inflammation in adipose tissue^3,4^. Recent evidence indicates that this persistent inflammation in obese adipose tissue is characterized by an unbalanced production of pro-inflammatory versus anti-inflammatory and pro-resolving mediators^5–7^. Among the latter, resolvins are the most widely known specialized pro-resolving mediators (SPMs) and they play a prominent role in inflammation because of their ability to expedite resolution with minimal damage to the surrounding tissue (reviewed in 8). Indeed, a deficit in SPM levels interferes with the timely resolution of inflammation as recently demonstrated in patients with chronic metabolic diseases and experimental models of obesity^7,9–12^. Although the biological actions of SPMs have been a subject of ample research, whether these lipid mediators and their role in the resolution process are influenced by genetic factors is currently unknown.

In the current study, we identified a functional SNP in the gene encoding for ERV1/ChemR23, the receptor that recognizes the anti-inflammatory and pro-resolving mediator resolvin E1 (RvE1), a SPM endogenously derived from the long-chain highly-unsaturated fatty acid, eicosapentaenoic acid (EPA)^13,14^. In particular, in this study we provide evidence that in comparison to obese individuals carrying the ancestral allele T, those individuals carrying the C variant in the ERV1/ChemR23 rs1878022 SNP exhibit a higher expression of this receptor in visceral adipose tissue, a reduced degree of adipose tissue inflammation and hepatic insulin resistance and significantly lower levels of circulating inflammatory cytokines and chemokines. In addition, LPS-stimulated leukocytes from obese individuals carrying the C variant were more responsive to RvE1, suggesting that this variant enhances RvE1-induced anti-inflammatory responses. The role of ERV1/ChemR23 in the regulation of the inflammatory tone was confirmed in mice lacking this receptor (ChemR23^−/−^), which, as compared to wild-type (WT) mice, displayed a greater degree of inflammation in visceral adipose tissue, liver tissue and peritoneal macrophages. Altogether, our data provide evidence of the influence of genetic factors on SPM actions and resolution of inflammation in patients with obesity.

## Results

The demographic and clinical characteristics of the patient cohorts are shown in Table 1. One hundred one out of 200 participants were morbidly obese and 68% were women. The obese cohort was significantly younger than the non-obese group (63 ± 3 vs. 45 ± 1 years, P < 0.001). Non-obese patients had a body mass index (BMI) of 26.4 ± 0.9 kg/m^2^ whereas patients from the obese cohort had a significantly increased BMI (45.3 ± 0.7 kg/m^2^). There were no statistically significant differences in serum glucose, triglycerides (TG), total cholesterol, gamma-glutamyl transferase (GGT), alanine aminotransferase (ALT) and aspartate aminotransferase (AST) levels between the two study cohorts (Table 1). No statistical significant differences were observed in blood leukocyte, monocyte and platelet counts (Table 1).

**Table 1:** Clinical characteristics and anthropometric measurements of the study participants.

	**Non-obese**	**Obese**
	n = 99	n = 101
♂/♀ (%)	42/58	32/68
Age (years)	63 ± 3	45 ± 1*
BMI (kg/m^2^)	26.4 ± 0.9	45.3 ± 0.7*
Glucose (mg/dL)	112.6 ± 13.0	119.4 ± 5.6
Triglycerides (mg/dL)	119.8 ± 28.1	133.7 ± 5.2
Cholesterol (mg/dL)	168.7 ± 21.4	189.9 ± 3.7
GGT (IU/L)	36.0 ± 9.0	39.2 ± 5.7
ALT (IU/L)	22.7 ± 7.4	29.8 ± 2.3
AST (IU/L)	20.3 ± 4.5	23.4 ± 1.4
Leukocytes (10^9^/L)	7.9 ± 0.6	8.4 ± 0.2
Monocytes (10^9^/L)	0.4 ± 0.1	0.5 ± 0.1
Platelets (10^9^/L)	273.9 ± 19.2	306.6 ± 8.1

BMI: body mass index; ALT: alanine aminotransferase; AST: aspartate aminotransferase.

Non-obese individuals (BMI < 29.9), Obese subjects (BMI > 29.9).

Normal reference values: glucose 65–110 mg/dL, triglycerides 50–150 mg/dL, cholesterol 148–247 mg/dL, GGT, ALT and AST 5–40 IU/L, leukocytes 4.5–10.5 × 10^9^/L, monocytes 0.14–0.72 × 10^9^/L and platelets 150–400 × 10^9^/L.

Results are expressed as mean ± SEM.

*P < 0.001 versus Non-obese.

Using TaqMan® allelic discrimination we first analyzed seven SNPs in genes involved in the regulation of the inflammatory response: interleukin (IL)-1β (rs1143634), IL-6 (rs1800795), signal transducer and activator of transcription 3 (STAT3, rs8069645), Janus kinase 2 (JAK2) (rs7849191), IL-10 (rs1800871), suppressor of cytokine signaling 3 (SOCS3, rs8064821) and ERV1/ChemR23 (rs1878022). These SNPs have previously been associated with inflammation or insulin resistance in the setting of metabolic, hepatic or proliferative disorders (Supplementary Table 1). All the selected candidate gene variants had minor allele frequencies (MAF) consistent with those reported in the HapMap project CEPH-CEU (Utah Residents with Northern and Western European Ancestry) (Table 2). The distribution of genotypes for all the SNPs was consistent with Hardy-Weinberg equilibrium (Table 2). Single locus analysis under the dominant inheritance model in the whole group of patients of the study identified a minor allele variant (C) in the ERV1/ChemR23 DNA gene sequence in association with the cohort of obese individuals (Table 3). This SNP (rs1878022) is an intronic variant located within ERV1/ChemR23 on human chromosome 12 (Supplementary Fig. 1). This gene encodes for a seven transmembrane G-protein receptor that binds the specialized pro-resolving lipid mediator RvE1^14^ as well as chemerin, a protein that participates in numerous cellular processes such as adipogenesis^15^. No other SNP studied was detected in association with the obese cohort (Table 3). TaqMan®SNP genotyping results of the ERV1/ChemR23 rs1878022 SNP were confirmed by Sanger DNA sequencing (Supplementary Fig. 2A). The genotype analysis revealed that the CC and TC genotypes were more frequent in obese individuals whereas the TT genotype was more frequent in non-obese individuals (Supplementary Fig. 2B).

**Table 2:** Common allele frequency, minor allele frequency (MAF), reported MAF and Hardy-Weinberg equilibrium for the SNPs genotyped in the study.

Gene (SNP ID.)	Common allele frequency	MAF	Reported MAF*	Χ^2^ value
IL-1β (rs1143634)	G (0.81)	A (0.19)	A (0.13)	0.975
IL-6 (rs1800795)	G (0.81)	C (0.19)	C (0.14)	0.072
STAT3 (rs8069645)	A (0.69)	G (0.31)	G (0.33)	0.547
JAK2 (rs7849191)	T (0.57)	C (0.43)	C (0.49)	0.113
IL-10 (rs1800871)	G (0.59)	A (0.41)	A (0.43)	1.309
SOCS3 (rs8064821)	C (0.81)	A (0.19)	A (0.20)	2.623
ERV1/ChemR23 (rs1878022)	T (0.69)	C (0.31)	C (0.32)	3.397

*Data from the HapMap project CEPH-CEU (Utah Residents with Northern and Western European Ancestry). χ^2^ value = 3.841 (1 degree of freedom, p value < 0.05).

**Table 3:** Analysis of association for each SNP with the cohort of obese individuals according to dominant, recessive and overdominant inheritance models.

**Gene and SNP**	**Inheritance Model**		**Odds ratio**	**CI 95%**	**P value**
IL-10 (rs1800871)	Dominant	GG/GA+AA	1.384	(0.793–2.481)	n.s.
	Recessive	AA/GG+GA	1.894	(0.672–5.339)	n.s.
	Overdominant	GA/GG+AA	1.148	(0.648–2.032)	n.s.
IL-6 (rs1800795)	Dominant	GG/GC+CC	1.080	(0.618–1.886)	n.s.
	Recessive	CC/GG+GC	0.495	(0.216–1.133)	n.s.
	Overdominant	GC/GG+CC	1.523	(0.865–2.683)	n.s.
STAT3 (rs8069645)	Dominant	AA/AG+GG	1.236	(0.704–2.170)	n.s.
	Recessive	GG/AA+AG	0.886	(0.371–2.114)	n.s.
	Overdominant	AG/AA+GG	1.353	(0.739–2.478)	n.s.
SOCS3 (rs8064821)	Dominant	CC/CA+AA	0.579	(0.298–1.125)	n.s.
	Recessive	AA/CC+C	0.310	(0.061–1.574)	n.s.
	Overdominant	ACA/CC+AA	0.699	(0.345–1.415)	n.s.
ERV1/ChemR23 (rs1878022)	Dominant	TT/TC+CC	1.704	(0.965–3.010)	<0.05
	Recessive	CC/TT+TC	1.355	(0.705–2.604)	n.s.
	Overdominant	TC/TT+CC	1.380	(0.770–2.474)	n.s.
JAK2 (rs7849191)	Dominant	TT/TC+CC	0.875	(0.402–1.904)	n.s.
	Recessive	CC/TT+TC	0.816	(0.459–1.450)	n.s.
	Overdominant	TC/TT+CC	1.130	(0.648–1.968)	n.s.
IL-1β (rs1143634)	Dominant	GG/GA+AA	0.749	(0.421–1.331)	n.s.
	Recessive	AA/GG+GA	0.978	(0.352–2.718)	n.s.
	Overdominant	GA/GG+AA	0.726	(0.393–1.340)	n.s.

**Figure 1:**
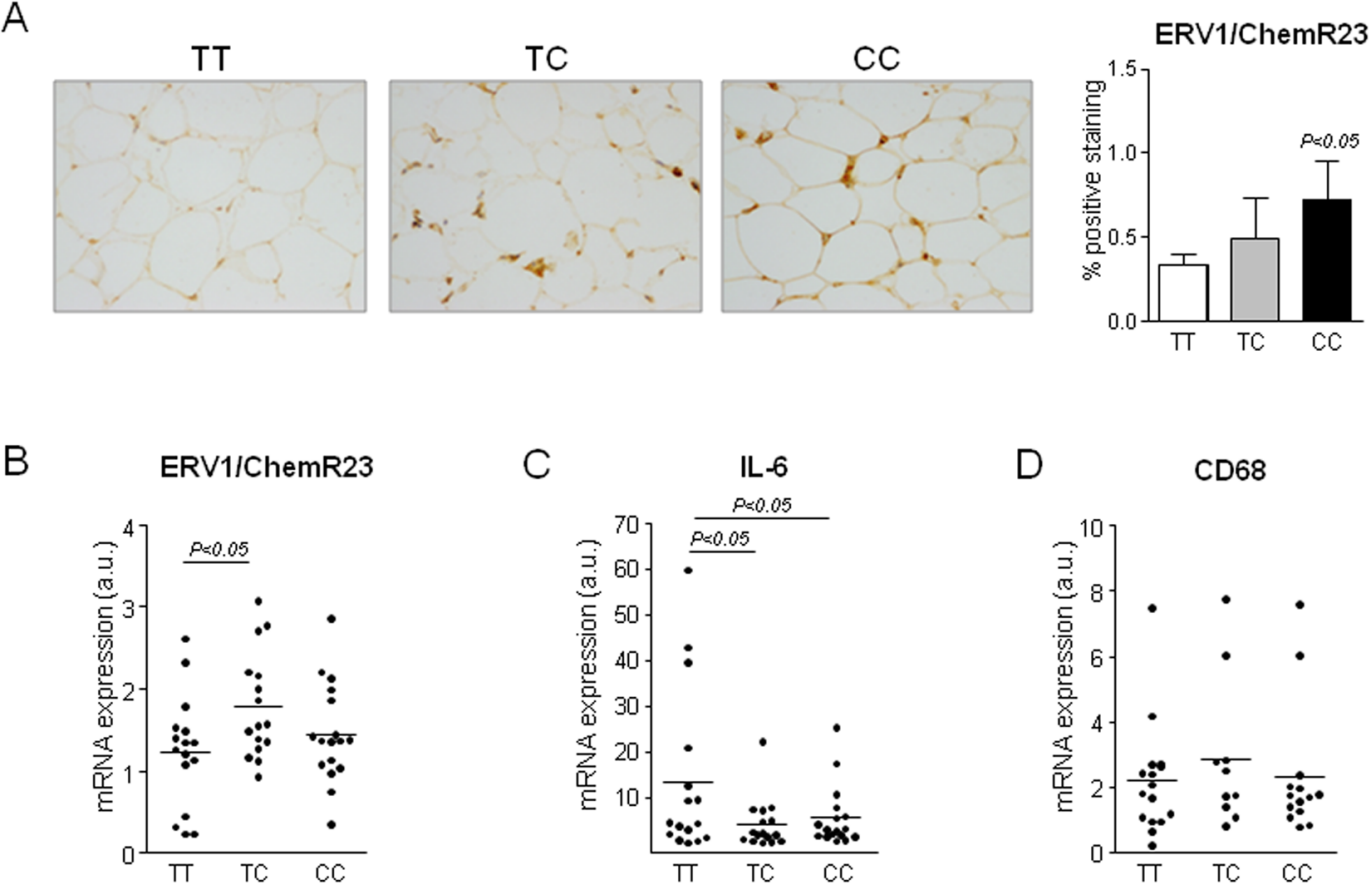
Overexpression of ERV1/ChemR23 in omental adipose tissue from obese individuals carrying the C allele. (**A**) Immunohistochemical staining of ERV1/ChemR23 protein in omental adipose tissue of obese patients carrying the TT (n = 16), TC (n = 16) and CC (n = 17) genotypes. Representative images with a 200X magnification are shown (left). Quantification of the percentage of the positive staining area is shown on the right. (**B**) Real-time PCR analysis of ERV1/ChemR23 mRNA expression. (**C**) IL-6 mRNA expression (**D**) CD68 mRNA expression. The data represent the mean ± SEM.

We next determined whether the ERV1/ChemR23 variant had a functional role in omental adipose tissue from obese individuals. The morphometric assessment of ERV1/ChemR23 protein levels as detected by immunohistochemistry revealed increased positive staining for this receptor in adipose tissue from obese patients carrying the C allele, reaching statistical significance in individuals that were homozygous (CC) (Fig. 1A). The adipose tissue expression of ERV1/ChemR23 at the mRNA level was significantly higher in heterozygotes of obese patients carrying the C allele (Fig. 1B). Notably, IL-6, a marker of inflammation, was significantly reduced in omental adipose tissue from obese individuals carrying the C allele (Fig. 1C). We were able to confirm the inverse relationship between the expression of ERV1/ChemR23 and IL-6 in 3T3-L1 adipocytes, which levels of ERV1/ChemR23 expression increase during the process of differentiation (Supplementary Fig. 3). Of interest, no differences in CD68, a macrophage surface marker, were observed in omental adipose tissue from obese individuals carrying the C allele (Fig. 1D), suggesting that variations in the degree of inflammation associated with the ERV1/ChemR23 variant were probably related to changes in the expression of inflammatory genes rather than to the number of infiltrated macrophages.

**Figure 2:**
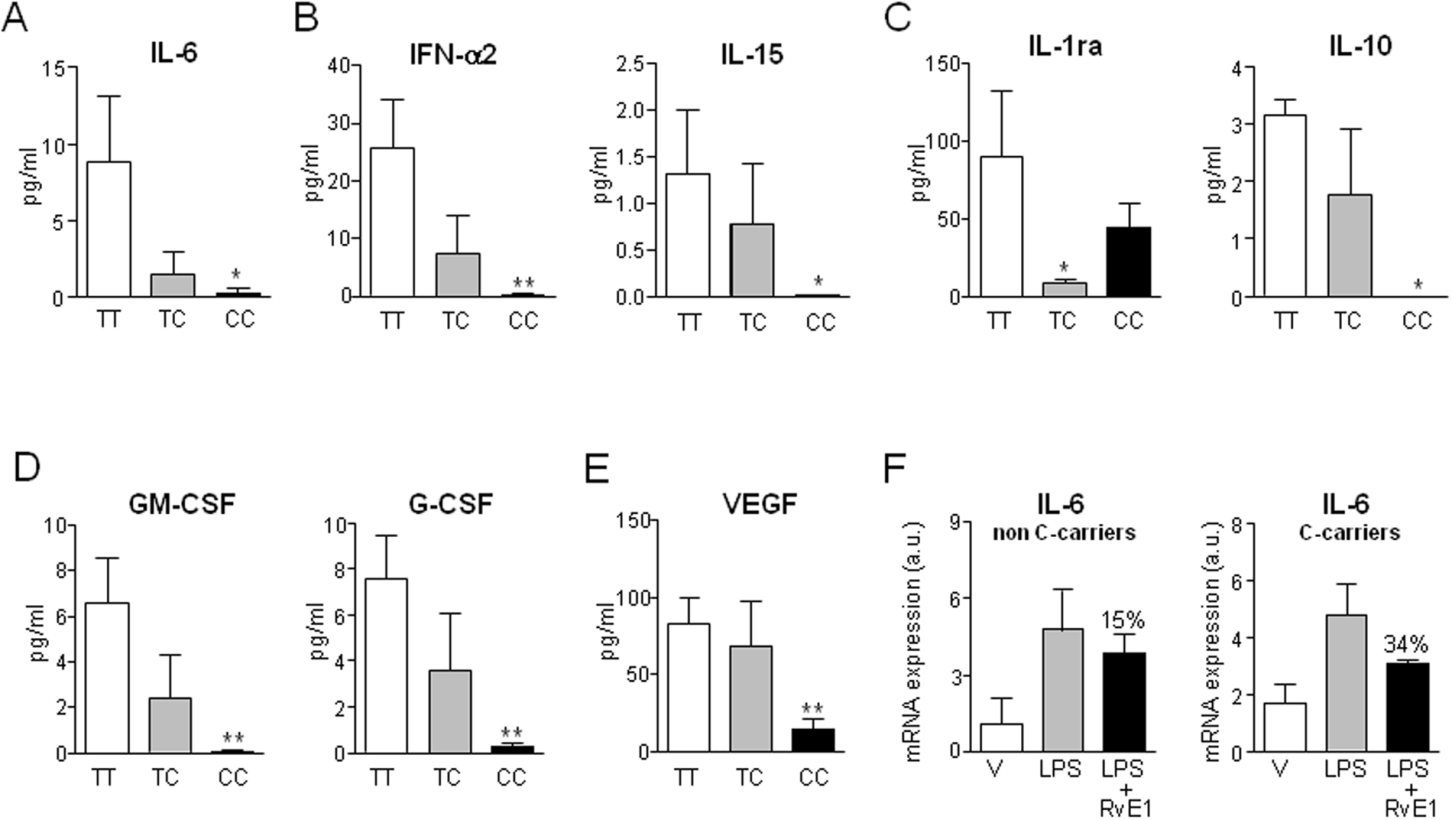
Circulating levels of cytokines in obese individuals carrying the different ERV1/ChemR23 genotypes. Plasma levels of IL-6 (**A**), IFN-α2, IL-15 (**B**), IL-1ra, IL-10 (**C**), GM-CSF and G-CSF (**D**), and VEGF (**E**) determined by the Luminex assay according to the ERV1/ChemR23 SNP genotype. (**F**) IL-6 expression in polymorphonuclear leukocytes isolated from non-C carrier and C-carrier individuals incubated with LPS (100 ng/ml) in the absence or presence of RvE1 (10 nM) for 2 h. The data represent the mean ± SEM of the individuals with TT (n = 16), TC (n = 16) and CC (n = 17) genotypes. *p < 0.05 and **p < 0.001 versus TT.

We next explored whether changes in the degree of adipose tissue inflammation in obese patients carrying the different ERV1/ChemR23 rs1878022 genotypes were mirrored by differential circulating levels of cytokines. As shown in Fig. 2A, obese patients carrying the minor C allele had lower plasma IL-6 levels than those carrying the TT genotype, reaching statistical significance in homozygous CC individuals. Other inflammatory and immunomodulatory cytokines such as interferon alpha-2 (IFN-α2) and IL-15 were also significantly reduced in plasma from CC individuals (Fig. 2B). Of interest, decreased levels of anti-inflammatory (interleukin-1 receptor antagonist (IL-1ra) and IL-10) (Fig. 2C), hematopoietic (granulocyte/macrophage colony stimulating factor (GM-CSF) and granulocyte colony stimulating factor (G-CSF)) (Fig. 2D) and pro-angiogenic (vascular endothelial growth factor (VEGF)) (Fig. 2E) cytokines were also seen in obese patients carrying the C allele. No differences were found in IL-8 and monocyte chemoattractant protein-1 (MCP-1) (Supplementary Fig. 4). In view of these findings, we hypothesized that circulating leukocytes from individuals carrying the C variant in the ERV1/ChemR23 receptor, which recognizes the pro-resolving mediator RvE1, would exhibit a more favorable anti-inflammatory environment than leukocytes from non-C carriers. To address this, we triggered LPS-induced inflammation in leukocytes from C-carrier and non-C carrier individuals in the presence or absence of RvE1 and quantified the inflammatory response by means of IL-6 expression. As shown in Fig. 2F, leukocytes from C-carrier patients were more sensitive to RvE1 and showed a higher capacity to attenuate the LPS induction of IL-6 than those isolated from non-C carriers (∼34% vs 15% reduction).

**Figure 3:**
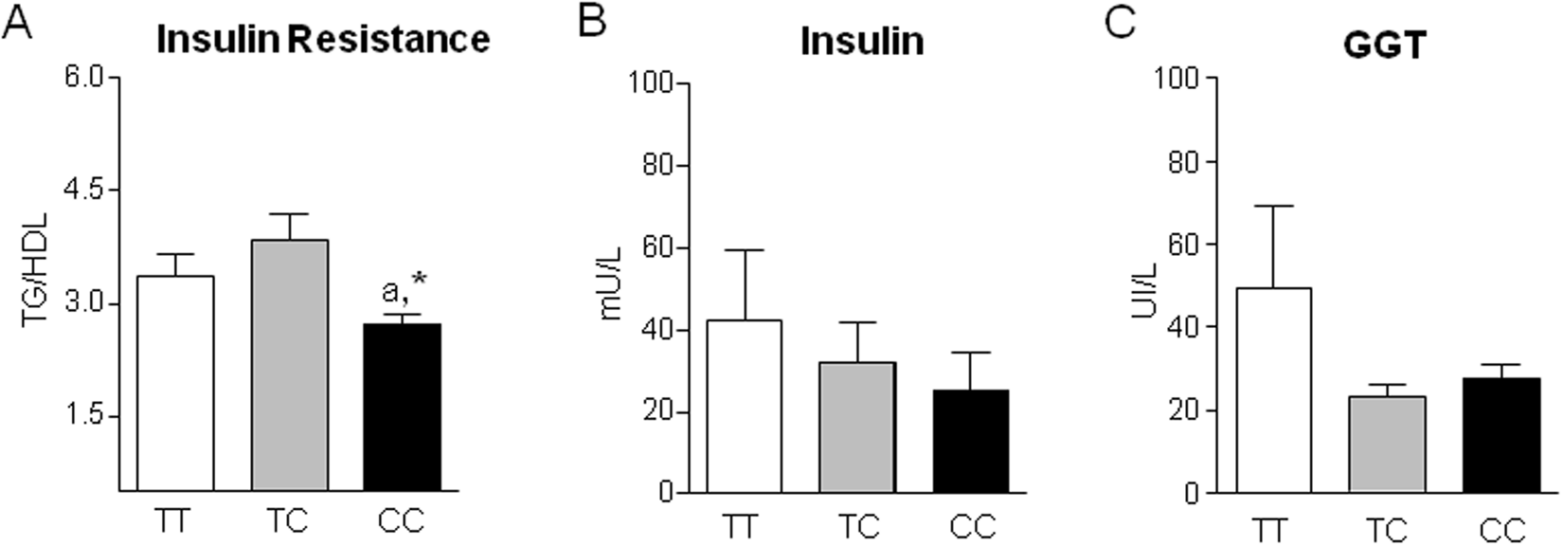
Individuals with the homozygous C variant in the ERV1/ChemR23 SNP show reduced insulin resistance. (**A**) TG to the high-density lipoprotein cholesterol (HDL-c) ratio. (**B**) Serum insulin. (**C**) Serum GGT levels. The data represent the mean ± SEM of the individuals with TT (n = 16), TC (n = 16) and CC (n = 17) genotypes. *p < 0.01 versus TC and a, p < 0.05 versus TT.

Obesity-induced inflammation is a well-established risk factor for metabolic comorbidities, such as insulin resistance and non-alcoholic fatty liver disease. As shown in Fig. 3A, obese patients carrying the minor C allele in homozygosity exhibited a significantly reduced TG to the high-density lipoprotein cholesterol (HDL-c) ratio, a surrogate marker of insulin resistance and a predictor of incident fatty liver and increased risk for cardiovascular disease in human subjects independently of obesity^16–19^. Plasma insulin and GGT showed a trend towards reduced levels in patients carrying the minor C allele (Fig. 3B and C). Together, these findings identify obese patients carrying the minor C allele as a subset of subjects with a lower-risk of metabolic syndrome.

**Figure 4:**
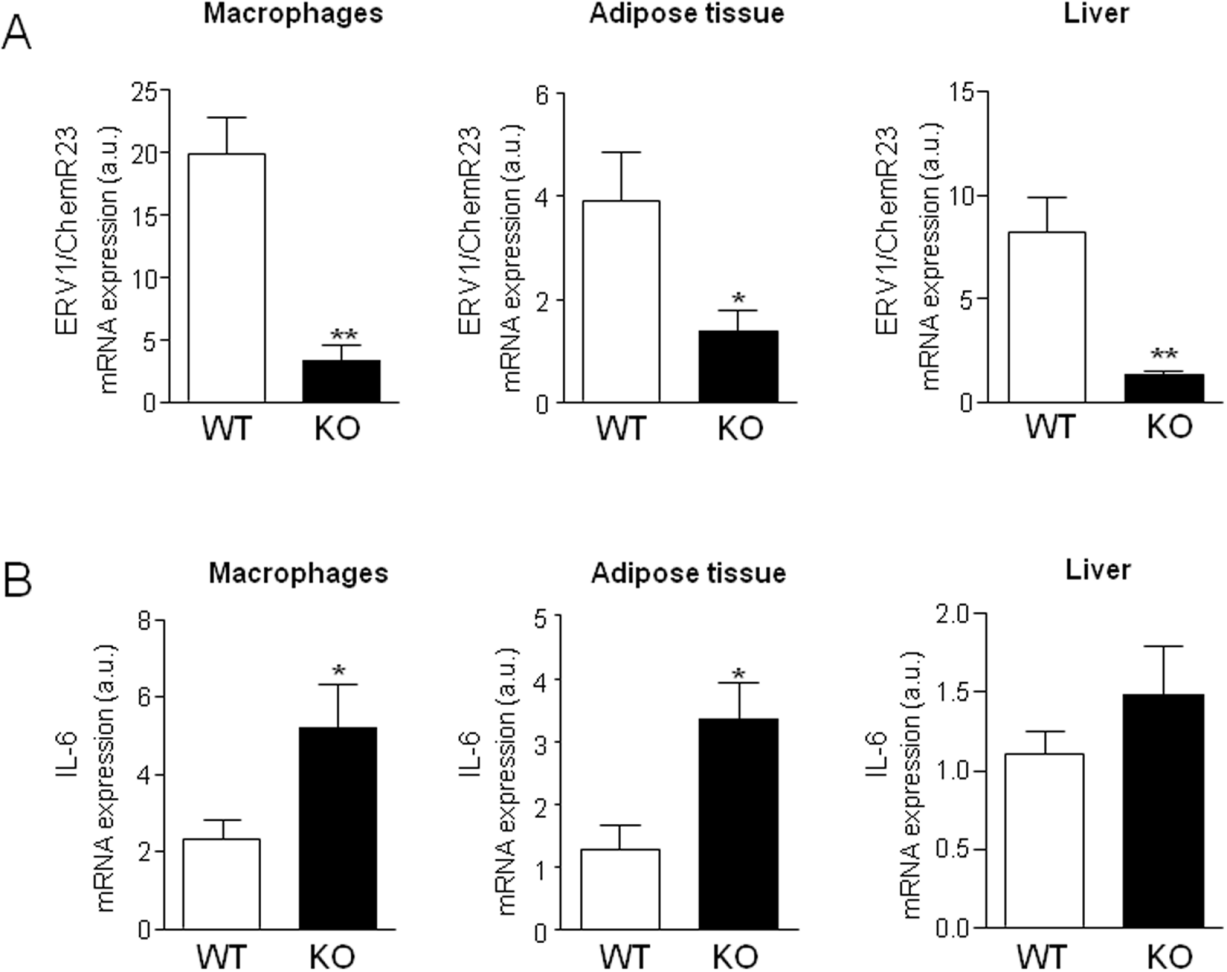
Mice lacking ERV1/ChemR23 display increased IL-6 expression. (**A**) ERV1/ChemR23 mRNA expression in visceral adipose tissue, peritoneal macrophages and liver from wild-type (WT) and KO mice. (**B**) IL-6 mRNA expression in these tissues from WT and KO mice. The data represent the mean ± SEM of WT (n = 12) and KO (n = 12) mice. *p < 0.05 and **p < 0.01 versus WT.

**Figure 5:**
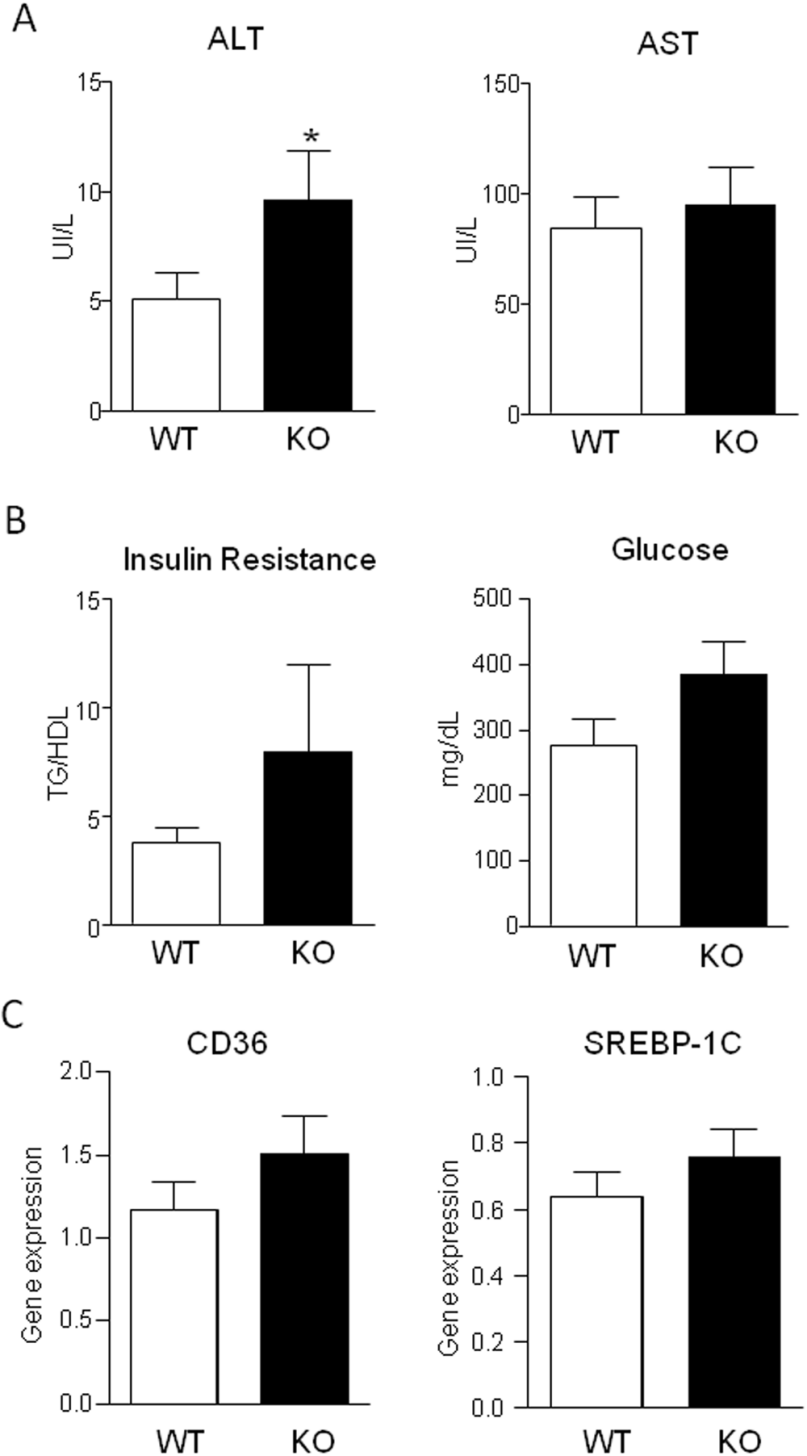
Mice lacking ERV1/ChemR2 (KO) show increased liver injury. (**A**) Serum levels of AST and ALT in wild-type (WT, n = 12) and KO (n = 12) mice. (**B**) Serum TG to the high-density lipoprotein cholesterol (HDL-c) ratio in these mice. (**C**) Hepatic expression of CD36 and SREBP-1c in WT and KO mice. The data represent the mean ± SEM. *p < 0.01 versus WT.

To confirm that changes in the expression of ERV1/ChemR23 are linked to variations in the degree of systemic and tissue inflammation we next explored the inflammatory phenotype of mice lacking ERV1/ChemR23 (ERV1/ChemR23^−/−^ mice). As compared to WT mice, no differences were observed in ERV1/ChemR23^−/−^ mice with respect to body weight and white adipose tissue and liver to body weight ratios (Supplementary Table 2). No changes were seen in total serum cholesterol, HDL-c, low-density lipoprotein cholesterol (LDL-c) and TGs (Supplementary Table 2). As expected, mice lacking ERV1/ChemR23 showed decreased expression of this receptor in peritoneal macrophages, visceral adipose tissue and liver (Fig. 4A). Compared to their WT controls and consistent with the view that ERV1/ChemR23 is a key regulator of inflammation, ChemR23^−/−^ mice had significantly increased expression of the pro-inflammatory cytokine IL-6 in peritoneal macrophages and adipose tissue (Fig. 4B). MCP-1 was also up-regulated in adipose tissue of ERV1/ChemR23^−/−^ mice (Supplementary Fig. 5). Expression of IL-6 were slightly higher in livers from ERV1/ChemR23^−/−^ mice, although differences did not reach statistical significance (Fig. 4B). The absence of changes in hepatic IL-6 in ChemR23^−/−^ mice were confirmed at the protein level (Supplementary Fig. 6). ERV1/ChemR23^−/−^ mice had increased levels of ALT, a surrogate serum marker of liver injury, and showed an increasing trend in insulin resistance and serum glucose levels (Fig. 5A and B). However, no evidence of steatosis was observed in these mice, since the hepatic expression of CD36 and SREBP-1c, two genes invariably up-regulated during the steatotic process, remained unchanged (Fig. 5C).

## Discussion

Inflammation plays a critical role in host defense against invasive pathogens and tissue and wound repair. Inflammation not only occurs in response to pathogens but can also be induced without active infection (sterile inflammation). This is the case of obesity, a condition in which the immune system is engaged in low-grade inflammatory response in several insulin-sensitive tissues, especially adipose tissue^20^. The stressors of this inflammatory response are diverse including over-nutrition, high-levels of lipids, free fatty acids and glucose, oxidative stress and hypoxia secondary to the expansion of adipose tissue volume^21^. A particular feature of obesity-induced inflammation is that it is chronic and of low intensity^20^. Importantly, this state of chronic low-grade inflammation is central in the pathogenesis of obesity-associated comorbidities such as insulin resistance^3,4^. For example, insulin resistance closely correlates with visceral obesity, ectopic fat deposition in muscle and liver, hypertension, dyslipidemia, endothelial dysfunction and elevated levels of adipokines such as TNFα, IL-6 and leptin^22,23^. This inflammatory environment progresses to an overproduction of reactive oxygen species and adipokines, which chronify the inflammatory response ultimately leading to obesity-related complications^3,4^.

Given that persistent unresolved inflammation is detrimental to the host, higher organisms have evolved protective mechanisms to ensure resolution of the inflammatory response in a specific time-limited manner^24^. Among the mechanisms that facilitate resolution, the biosynthesis of SPMs, a class of endogenous lipid mediators which includes, among others, resolvins, protectins and maresins generated from the omega-3 fatty acids EPA and docosahexaenoic acid (DHA), has been described to efficiently resolve inflammation with minimal damage to the surrounding tissue^8,25^. In particular, RvE1 is formed from EPA during the resolution phase of acute inflammation via cell-cell interactions such as endothelial cell-leukocyte interactions^13,14^. RvE1 biosynthesis involves cells bearing acetylated cyclooxygenase (COX)-2 and cells that possess 5-lipoxygenase (5-LOX), although it can also be generated by cytochrome P450 processing of EPA^26^. RvE1 blocks and counterregulates the production of inflammatory mediators, inhibits polymorphonuclear leukocytes transendothelial migration and stimulates macrophages to enhance phagocytosis and clearance of apoptotic leukocytes^27,28^. RvE1 actions are mediated by its binding to ERV1/ChemR23, a G-protein coupled receptor expressed in monocytes/macrophages, immature dendritic cells, and adipocytes^14,28^. Therefore, our observation that cells and tissues from patients carrying the C variant in the rs1878022 polymorphism of the ERV1/ChemR23 gene exhibit a more favorable anti-inflammatory environment in the context of higher expression of this receptor can be explained by the fact that these individuals are more responsive to the anti-inflammatory actions of RvE1. Indeed, our data demonstrate that leukocytes from C carriers show enhanced responsiveness to RvE1 in blocking LPS-induced inflammation than those from non-C carriers. Similar results were obtained in differentiating adipocytes in which higher expression of the RvE1 receptor ERV1/ChemR23 inversely correlated with lower expression of the inflammatory cytokine IL-6. However, it must be considered that ERV1/ChemR23 has other ligands, such as the peptide chemerin, which possesses antimicrobial and immunomodulatory properties^29,30^. Consequently, we cannot exclude mechanism other that RvE1 such as that described by Luangsay *et al*.^31^, who described that the ERV1/ChemR23 receptor mediates anti-inflammatory actions of chemerin in a lung disease model.

Several SNPs located within the ERV1/ChemR23 DNA sequence have been identified by genome-wide association studies demonstrating the impact of these variants on the natural course of multifactorial diseases. For example, rs17040430 has been associated with mental disorders in a cohort of 4436 patients with schizophrenia and/or bipolar disorder^32^. Another ERV1/ChemR23 SNP, rs107291463, has been strongly associated with the development of erectile dysfunction after radiotherapy^33^. Finally, the SNP rs1878022, identified in the current study as a protective variant against obesity-induced inflammation, has previously been demonstrated to be associated with poor overall survival in non‐small cell lung cancer patients^34^. As far as we know, our study is the first investigation in which a genetic variant of ERV1/ChemR23 has been associated with a higher degree of inflammation in both visceral adipose tissue and systemic circulation. Indeed, our observation reinforces the idea that resolution of inflammation, as well as many other biological processes, is influenced by our genetic background. In a previous report, Simiele and collaborators reported the presence of a SNP variant in the promoter of the gene coding for formyl peptide receptor 2 (FPR2)/ALX, a G-protein-coupled receptor that binds the pro-resolving mediators lipoxin A_4_ and RvD1^35^. These investigators reported that individuals carrying this SNP had a reduced expression of the pro-resolving FPR2/ALX receptor and were more vulnerable to developing cardiovascular disease^35^. On the other hand, Kim and collaborators have recently reported a gene variant in FPR2/ALX that conferred significant protection to asthma patients against the development of aspirin exacerbated respiratory diseases, which in this case was associated with an increased protein expression of this pro-resolving receptor^36^. Finally, a previously unappreciated pattern of methylation that makes FPR2/ALX transcriptionally inaccessible, leading to a low expression of this pro-resolving receptor at both mRNA and protein level has recently been uncovered^37^. This latter finding suggests that the expression of pro-resolving receptors is not only dependent on genetic variants but is also regulated by epigenetic factors.

An interesting aspect of our study was that we were able to confirm our findings in obese individuals at experimental level describing a close relationship between ERV1/ChemR23 expression and the degree of inflammation in mice lacking the ERV1/ChemR23 receptor. In these mice, we confirmed that the expression of this receptor inversely correlates with the levels of inflammatory markers in visceral adipose tissue. Our findings are consistent with those by Demoor *et al*.^38^, who reported that mice lacking ERV1/ChemR23 exhibit an impaired resolution of cigarette smoke-induced inflammation. Moreover, our data point to the presence of insulin resistance in these mice, results which are consistent with those previously reported by Ernst *et al*.^39^ showing that ERV1/ChemR23 knockout mice are glucose intolerant. However, these findings do not agree with those from Rouger *et al*.^40^, who reported unaltered glucose tolerance in these mice.

In summary, the results of the present study provide evidence that a SNP variant in the ERV1/ChemR23 gene is associated with increased expression of this receptor and with a lower degree of tissue inflammation and circulating levels of cytokines and chemokines in a population of morbidly obese individuals. Importantly, patients carrying this SNP variant exhibit a lower risk of obesity-associated comorbidities such as insulin resistance. The inverse relationship between ERV1/ChemR23 expression and the degree of inflammation was confirmed in ERV1/ChemR23 knockout mice, which displayed a greater degree of inflammation in both visceral adipose tissue, liver and peritoneal macrophages. Collectively, our data indicate that the ERV1/ChemR23-RvE1 axis is susceptible to modulation by genetic changes, corroborating that the resolution of inflammation is also regulated at the DNA level.

## Material and Methods

### Study participants

One hundred one patients with morbid obesity (BMI > 30 kg/m^2^) undergoing laparoscopic bariatric surgery and 99 non-obese patients (BMI < 30 kg/m^2^) undergoing elective gastric surgery were included in the study. The BMI was calculated as mass/(height)^2^. Individuals with inflammatory bowel disease or cancer and obese patients with previous bariatric surgery were excluded from the study. Demographic, clinical data and drug use were collected from the electronic medical records of the patients. Venous blood samples (5 ml) were collected in EDTA-tubes and visceral (omental) adipose tissue samples were intra-operatively harvested with sharp dissection for genotyping and gene expression analysis, respectively. Harvested adipose tissue samples were weighed on a precision balance, washed twice with DPBS, cut into 100 mg pieces, placed in either 10% formalin or snap-frozen in liquid nitrogen and stored in Nunc® CryoTubes at −80 °C for further analysis. The study protocol (protocol #2012-7239) was approved by the Investigation and Ethics Committee of the Hospital Clínic and all methods were carried out in accordance with the guidelines and regulations dictated by this Committee. Written informed consent was obtained from all participants.

### Biochemical analyses and cell count

Serum concentrations of glucose, total cholesterol, HDL-c, TG, ALT, AST and GGT and blood leukocyte, monocyte and platelet counts were determined by standard laboratory procedures. LDL-c was calculated as (total cholesterol – HDL-c) – (TG/5).

### Assessment of insulin resistance

Insulin resistance was assessed by calculating the ratio between serum TG and HDL-c levels, as a surrogate marker of insulin resistance and a predictor of incident fatty liver and increased risk for cardiovascular disease in human subjects independently of obesity^16–19^.

### DNA extraction

Blood samples were centrifuged at 800 g for 15 min and genomic DNA was isolated from the buffycoat containing the nucleated cells using the Qiasymphony MIDI Kit in a Qiasymphony SP instrument (Qiagen, Hilden, Germany). In some patients, DNA was extracted from visceral adipose tissue using the Qiasymphony MINI Kit (Qiagen) for tissue samples.

### Allelic Discrimination

Seven SNPs from seven candidate genes involved in the inflammatory response were screened. SNP selection was based on the inclusion of genes related to the inflammatory process. Among these, the screening included SNPs in genes coding for pro-inflammatory (IL-1β, IL-6, STAT3 and JAK2), anti-inflammatory (IL-10 and SOCS3) and pro-resolving (ERV1/ChemR23) factors and signaling pathways. The selection was based on previously published results showing allelic associations between these polymorphisms and human diseases (Supplementary Table 1). SNPs were genotyped using TaqMan®SNP Genotyping assays in an ABI 7900HT Sequence Detection System (Applied Biosystems, Foster City, CA). The TaqMan®SNP Genotyping assay was based on an oligonucleotide (probe) labeled with a reporter fluorescent dye (FAM^TM^ or VIC^TM^) and a quencher dye (TAMRA^TM^), linked covalently to the 5′ and 3′ end, respectively. The TaqMan probes used for genotyping were: IL-10 rs1800871 (C_1747362_10), IL-6 rs1800795 (custom assay), STAT3 rs8069645 (C_30301828_10), SOCS3 rs8064821 (C_43672951_10), ERV1/ChemR23 rs1878022 (C_11698200_10), JAK-2 rs7849191 (C_2008287_10) and IL-1β rs1143634 (C_9546517_10). Reactions took place in 96-well plates in 25 µl of total volume containing 11.25 µl of genomic DNA (1.77 ng/µl), 12.5 µl of Master Mix and 1.25 µl of a mix of probes and primers. During PCR amplification, the probe specifically anneals between the forward and reverse primer sites, and the nuclease activity of Taq DNA polymerase cleaves the probe and frees the fluorescent dye allowing allele identification. Data were analyzed by Sequence Detector Software (SDS) version 2.1 (Applied Biosystems).

### DNA Sequencing

The TaqMan®SNP Genotyping results were confirmed by Sanger DNA sequencing. PCR products were purified using the ExcelaPure™ 96-Well ultrafiltration-based system (EdgeBio, Gaithesburg, MD) and sequencing reactions were performed with the BigDye Terminator v.3.1 (Applied Biosystems). Sequencing reactions were cleaned-up by removal of unincorporated dyes with the Performa® DTR system (EdgeBio) before electrophoresis analysis in an ABI Prism 3130xl Genetic Analyzer (Applied Biosystems).

### Immunohistochemistry analysis

Adipose tissue samples were routinely fixed in 10% buffered formalin, embedded in paraffin and sectioned into 2-mm-thick sections for immunohistochemistry of primary rabbit anti-human ERV1/ChemR23 antibody (dilution 1:100; LifeSpan Biosciences, Seattle, WA) and H&E counterstaining at the Pathology Department of the Hospital Clínic. Sections were visualized at x200 magnification in a Nikon Eclipse E600 microscope (Tokyo, Japan).

### RNA extraction, reverse transcription and real-time PCR analysis

Isolation of total RNA was performed from 70 mg (human) and 40 mg (mouse) of adipose tissue by phenol-chloroform extraction using the TRIzol reagent. Dry RNA pellets were resuspended into 20 μl of DEPC water and RNA concentrations were assessed in a NanoDrop-1000 spectrophotometer (NanoDrop Technologies, Wilmington, DE). Five-hundred ng of total RNA were taken for cDNA synthesis using the High-Capacity cDNA Archive Kit (Applied Biosystems). Validated and pre-designed TaqMan® Gene Expression Assays were used to quantify IL-6 (ID: Hs00985639_m1; Mm00446190_m1), ERV1/ChemR23 (ID: Hs01386064_m1**;** Mm02619757_s1), MCP-1 (ID: Mm00441242_m1) and CD68 (ID: Hs00154355_m1; Mm03047343_m1) using β-actin (ID: Hs99999903_m1; Mm02619580_g1) as endogenous control. Real-time PCR amplifications were carried out in an Applied Biosystems 7900HT Fast Real Time PCR System. The amount of target gene, normalized to β-actin and relative to a calibrator, was determined by the arithmetic formula 2^−ΔΔCt^ described in the Comparative Ct Method (http://docs.appliedbiosystems.com/pebiodocs/04303859.pdf). Data analysis was performed using RQ Manager 1.2 (Applied Biosystems).

### Assessment of cytokines and chemokines by Luminex xMAP technology

Levels of cytokines including IL-6, IL-8, IL-15, MCP-1, TNF-α, G-CSF, GM-CSF, IL-10, IL-1ra, VEGF and IFN-α2 were determined in 25 μl of plasma using a custom-made multiplexed bead-based immunoassay (Milliplex MAP Human Cytokine/Chemokine Magnetic Bead Panel; Merck Millipore, Billerica, MA) in a Luminex 100 Bioanalyzer (Luminex Corp., Austin, TX). Briefly, 25 μL of plasma were added to each well before the addition of 25 μL of premixed microbeads. The plate was incubated overnight at 4 °C with shaking, then washed and reincubated with 25 μL of detection antibody for 1 hour. The plate was washed again and incubated with 25 μL of streptavidin-phycoerythrin for 30 minutes. Finally, after 2 washes the beads were resuspended with 100 μL of sheath fluid and analyzed in the Luminex 100 system. The readouts were analyzed with the standard version of Milliplex Analyst software (Merck Millipore). A five-parameter logistic regression model was used to create standards curves (pg/ml) and to calculate the concentration of each sample.

### Measurement of IL-6 levels

Levels of IL-6 in homogenates from liver tissue were determined by a specific enzyme-linked immunoassay (Mouse IL-6 ELISA Kit; Abcam, ab46100).

### Isolation of human peripheral blood leukocytes

Human peripheral blood leukocytes were isolated from C carrier and non-C carrier individuals. Briefly, blood samples collected with EDTA were centrifuged at 200 g for 10 min, and sedimented cells were incubated with pre-warmed ammonium-chloride-potassium lysis buffer for 5 min at room temperature to remove red blood cells. Samples were then centrifuged at 400 g for 10 min and the supernatants decanted. The red blood cell lysis procedure was repeated twice for 10 min each, and the resultant pellet was finally washed with DPBS without calcium and magnesium. The isolated leukocytes were resuspended in RPMI 1640 medium containing penicillin (100 U/mL) and streptomycin (100 U/mL) and L-glutamine (4 mM) with 0.5% FBS. Leukocytes at a density of 3 × 10^6^ cells/mL were stimulated with LPS (100 ng/ml) for 2 hours at 37 °C (5% CO_2_) in the absence or presence of RvE1 (10 nM). At the end of the incubations, cells were centrifuged at 400 g for 10 minutes at 4 °C and pelleted cells were collected for RNA extraction, reverse transcription and real-time PCR analysis as described above.

### Studies in ERV1/ChemR23 gene-deficient mice

Male and female ERV1/ChemR23 knockout mice were obtained from Deltagen (San Mateo, CA). Heterozygous mice in the C57BL/6 background were interbred to generate homozygous ERV1/ChemR23 knockout mice (n = 12) and their WT controls (n = 12). All experimental protocols were approved by the Ethical Committee of Animal Experimentation of the University of Barcelona (authorization# 9362) in accordance with the guidelines set by the Direcció General de Polítiques Ambientals i Medi Natural of the Generalitat de Catalunya and the regulations of the European Union legislation. Genomic DNA from the ear was isolated using the Omni-Pure Tissue Genomic DNA System (Gene Link, Hawthorne, NY) following the manufacturer’s protocol and genotyped by PCR. Two different PCR reactions of 20 μL were performed for detection of endogenous (E) and targeted (T) alleles. Expected product sizes in base pairs (bp) and a schematic diagram of the ERV1/ChemR23 knockout construct are shown in Supplementary Fig. 7A. The PCR reactions contained 0.5 µM of primers (forward direction: 5′-TACAGCTTGGTGTGCTTCCTCGGTC-3′ (ChemR23 primer or E) and 5′-GGGTGGGATTAGATAAATGCCTGCTCT-3′ (Neo primer or T); reverse direction: 5′-TGATCTTGCACATGGCCTTCCCGAA-3′ (common E and T)), 0.2 mM dNTPs mix, 1.5 mM MgCl_2_, and 1 U Platinum Taq DNA Polymerase (Invitrogen, Carlsbad, CA). PCR cycle conditions were 15 min at 95 °C followed by 30 cycles of 20 s at 94 °C, 40 s at 62 °C and 1 min at 72 °C, and a final step of 10 min at 72 °C and then cooled to 4 °C. PCR bands were separated by electrophoresis in 2.5% LM Sieve agarose gels and visualized by GelRed^TM^ Nucleic Acid Gel Stain (Biotium, Hayward, CA) using a 100-bp DNA ladder marker (Invitrogen). Gel images of the resulting PCR products of a typical offspring composed by 3 wild-type, 5 heterozygous ERV1/ChemR23^+/−^ and 1 homozygous ERV1/ChemR23^−/−^ littermates, are shown in Supplementary Fig. 7B. The mice were housed in wood-chip bedding cages with 50%-60% humidity under a 12 h light/12 h darkness cycle with unlimited access to food and water. At 18 weeks of age, the mice were euthanized *via* ketamine/xylazine injection (i.p., 4:1), and visceral adipose tissue and liver were excised, rinsed in DPBS and snap-frozen in liquid nitrogen for RNA analyses. All animal studies were conducted in accordance with the Investigation and Ethics Committee criteria of the Hospital Clínic and European Union legislation.

### Isolation of mouse peritoneal macrophages

Isolation of peritoneal macrophages from ChemR23^+/−^, ChemR23^−/−^ and ChemR23^+/+^ mice was performed as described by Titos *et al*.^9^. Briefly, peritoneal macrophages were collected by peritoneal lavage with 7 ml ice-cold DPBS^−/−^ three days after the i.p. injection of 2.5 ml of 3% thioglycolate. The exudates were centrifuged at 500 g for 5 min at 4 °C and further resuspended in DMEM supplemented with penicillin (100 U/ml), streptomycin (100 mg/ml), 2 mM L-glutamine and 5% FBS. The cells (150,000 cells/well) were allowed to adhere on 6-wells culture plates over 2 h at 37 °C in a humidified 5% CO_2_ incubator. Non-adherent cells were removed by washing twice with DPBS^−/−^ and the remaining adherent cells were used for the experiments. Finally, the macrophages were washed twice and then collected in TRIzol reagent and kept at −80 °C for RNA extraction and gene expression analysis.

### Differentiation and incubation of 3T3-L1 adipocytes

Mouse 3T3-L1 cells were seeded onto six-well plates (250,000 cells per well) in DMEM supplemented with 10% (vol/vol) FBS, 100 U/mL penicillin/streptomycin and 4 mM L-glutamine in a humidified atmosphere of 5% CO2 at 37 °C and allowed to grow to confluence for 2 days. Some confluent 3T3-L1 cells were either differentiated with adipocyte induction medium or left undifferentiated. The adipocyte induction medium contained insulin (5 μg/mL), isobutylmethylxanthine (0.5 mM), dexamethasone (0.25 μM), penicillin/streptomycin (100 U/mL), and L-glutamine (4 mM) in DMEM supplemented with 10% FBS. After 2 days, the induced cells were cultured in continuation medium (5 μg/mL insulin) for 72 hours and then maintained in DMEM supplemented with 10% FBS until exhibiting an adipocyte phenotype at day 8 of differentiation.

### Statistical analysis

Study groups were tested for Hardy-Weinberg equilibrium and observed and expected allele and genotypic frequencies were compared by χ^2^ analysis. Sample size calculations were conducted using Quanto software version 1.2.4. Allele and genotype frequencies for each SNP were compared between the obese and control groups using a 2 × 2 contingency table and calculation of the odds ratio with a 95% confidence interval. The relation of dependence between the minor allele and obesity was studied by Spearman correlation for non-parametrical variables. Statistical analysis of anthropometric data and gene expression results was performed using the unpaired Student’s t test. The results were expressed as means ± SEM and the level of statistical significance was set at P < 0.05.

## Electronic supplementary material

Supplementary information
