# Supplementary material for: Association of a variant in the gene encoding for ERV1/ChemR23 with reduced inflammation in visceral adipose tissue from morbidly obese individuals
Source: Sci Rep. 2017 Nov 16;7:15724. doi: 10.1038/s41598-017-15951-z (PMC5691181; doi:10.1038/s41598-017-15951-z)
Supplement: Supplementary file 1 — Supplementary information [file 41598_2017_15951_MOESM1_ESM.pdf]

Association of a variant in the gene encoding for ERV1/ChemR23 with reduced inflammation in visceral adipose tissue from morbidly obese individuals

Cristina López-Vicario[1,3], Bibiana Rius[1], José Alcaraz-Quiles[1], Ana González-Périz[1], Ana Isabel Martínez-Puchol[1], Mireia Casulleras[1], Marta Duran-Güell[1], Ainitze Ibarzabal[2], Ricard Corcelles[2], Andrés Laguna-Fernández[5], Magnus Back[5], Esther Titos[1,3], Joan Clària[1,3,4]

[1]Department of Biochemistry and Molecular Genetics and [2]Department of Gastrointestinal Surgery, Hospital Clínic, IDIBAPS; [3]CIBERehd and [4]Department of Biomedical Sciences, University of Barcelona, Barcelona, Spain; [5]Centre for Molecular Medicine, Department of Medicine, Karolinska Institutet and Department of Cardiology, Karolinska University Hospital, Stockholm, Sweden.

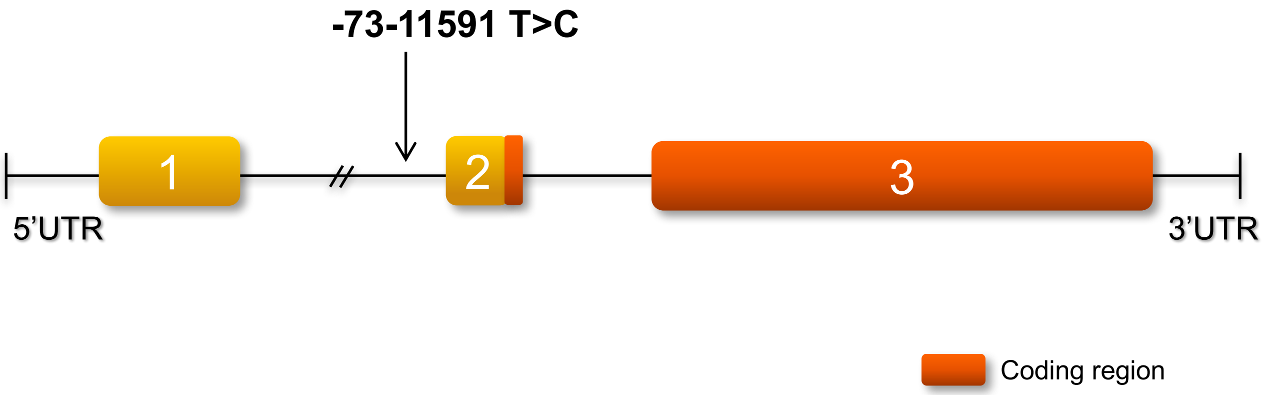

Supplementary Figure 1. Structure of the ERV1/ChemR23 gene. The ERV1/ChemR23 gene contains 3 exons and 2 introns. The SNP rs1878022 (nucleotide change from T to C) is located in the first intron of the gene. Transcription starts in exon 1. Coding region starts in the last codon of exon 2 and covers exon 3.

A

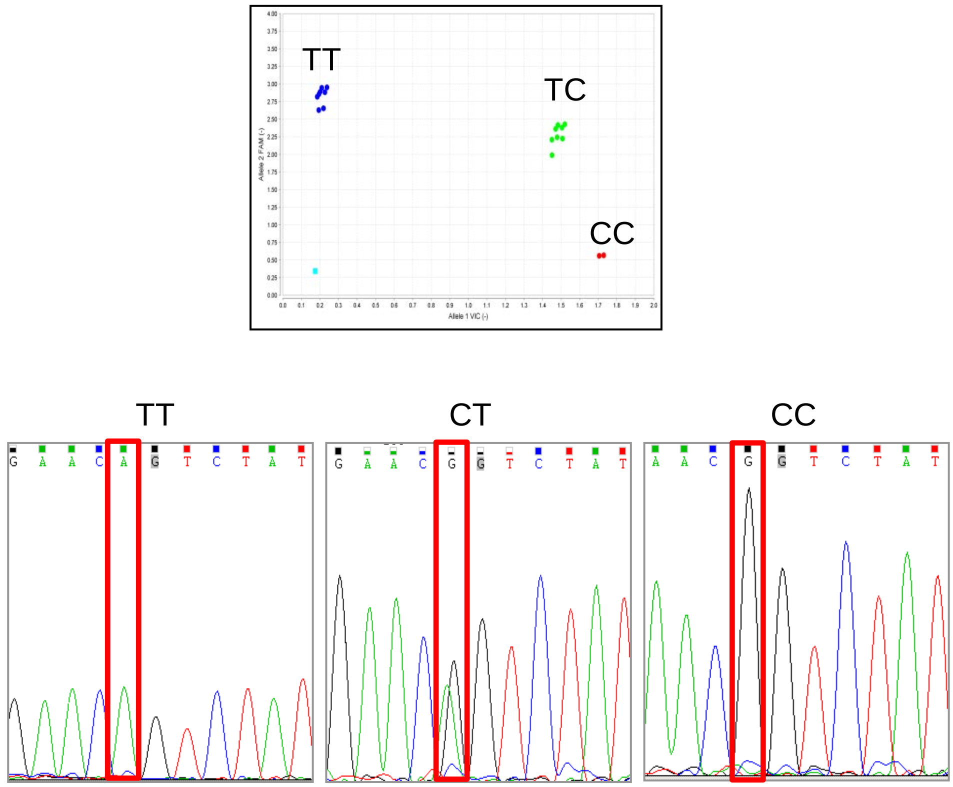

B

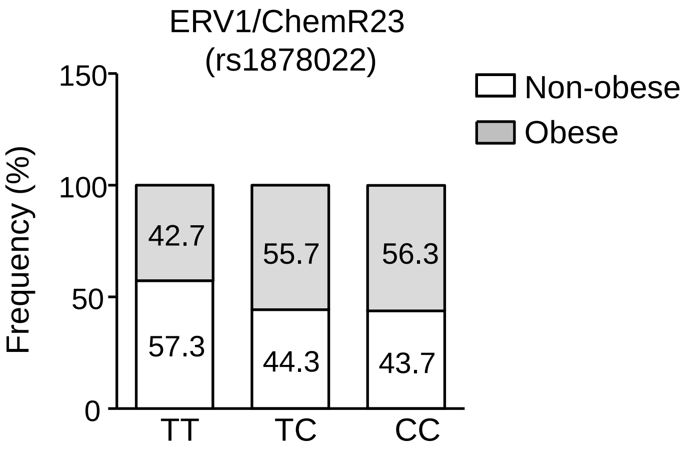

Supplementary Figure 2. (**A**) Representative data of the analysis of the ERV1/ChemR23 rs1878022 SNP by the TaqMan SNP genotyping assay (top) and Sanger sequencing (bottom). (**B**) Frequency of the three ERV1/ChemR23 rs1878022 TT (n=82), TC (n=70) and CC (n=48) genotypes in obese and non-obese individuals.

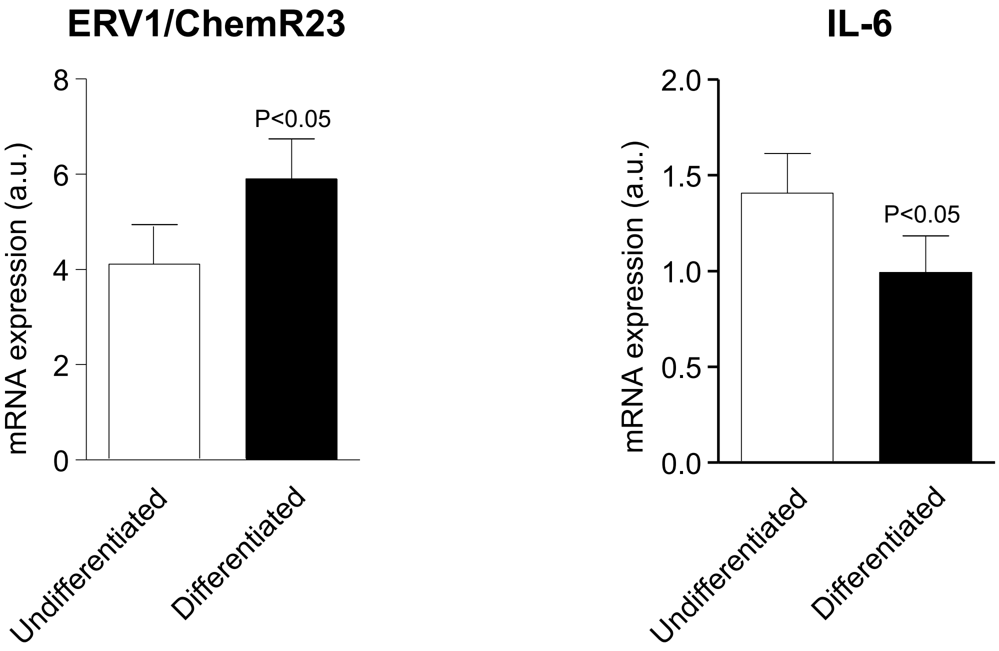

Supplementary Figure 3. ERV1/ChemR23 and IL-6 mRNA expression in undifferentiated and differentiated 3T3-L1 adipocytes. The data represent the mean ± SEM from 3 different experiments in duplicate.

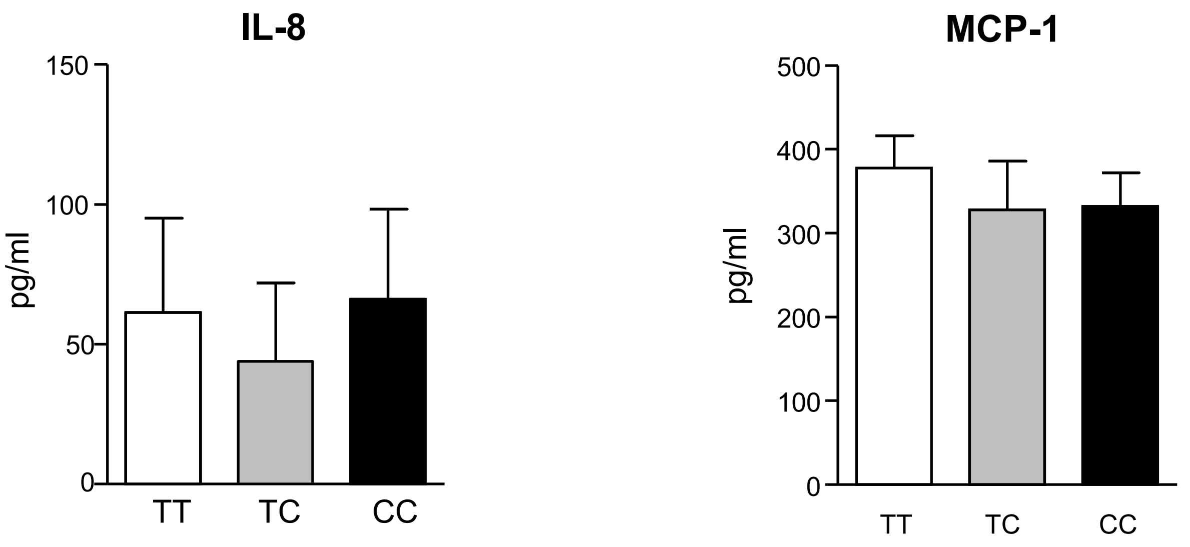

Supplementary Figure 4. Circulating levels of IL-8 and MCP-1 in obese individuals as determined by Luminex assay. The data represent the mean ± SEM of the individuals with TT (n=16), TC (n=16) and CC (n=17) genotypes.

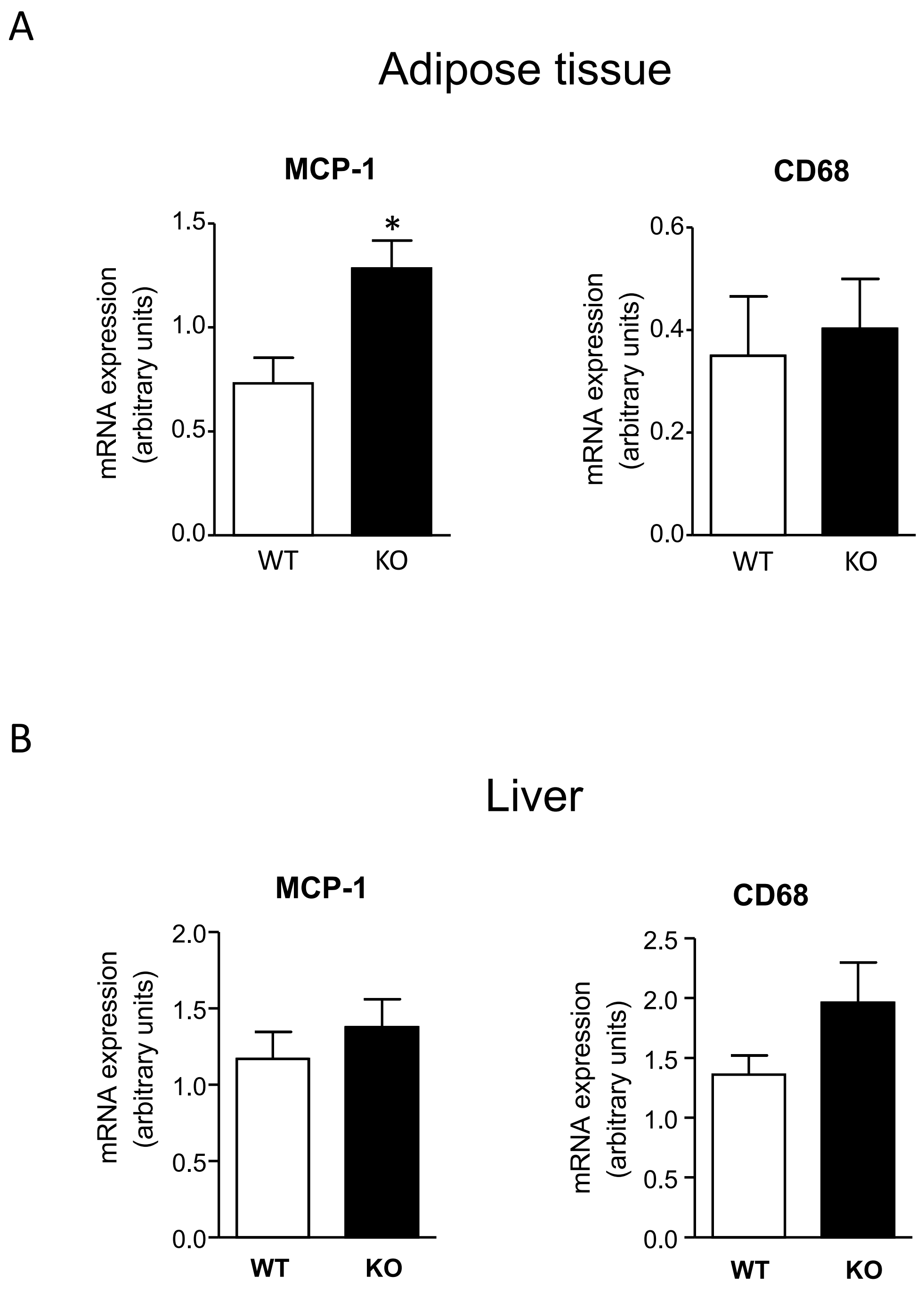

Supplementary Figure 5. RT-PCR results of MCP-1 and CD68 inflammatory markers in (A) adipose tissue and (B) liver from WT and KO mice. The data represent the mean ± SEM of WT (n=12) and KO (n=12) mice. *, $p<0.05$ versus WT.

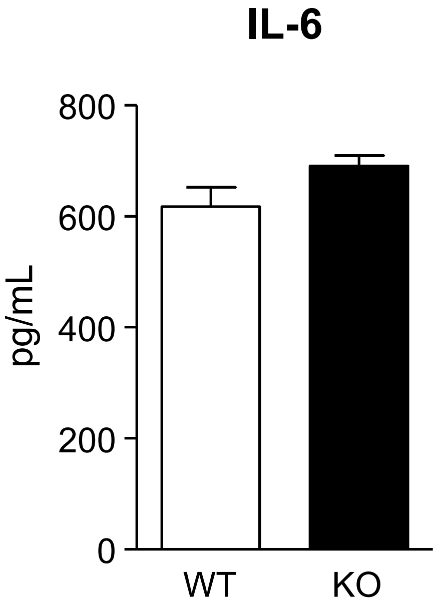

Supplementary Figure 6. IL-6 levels in livers from WT and KO mice. The data represent the mean ± SEM of WT (n=12) and KO (n=12) mice.

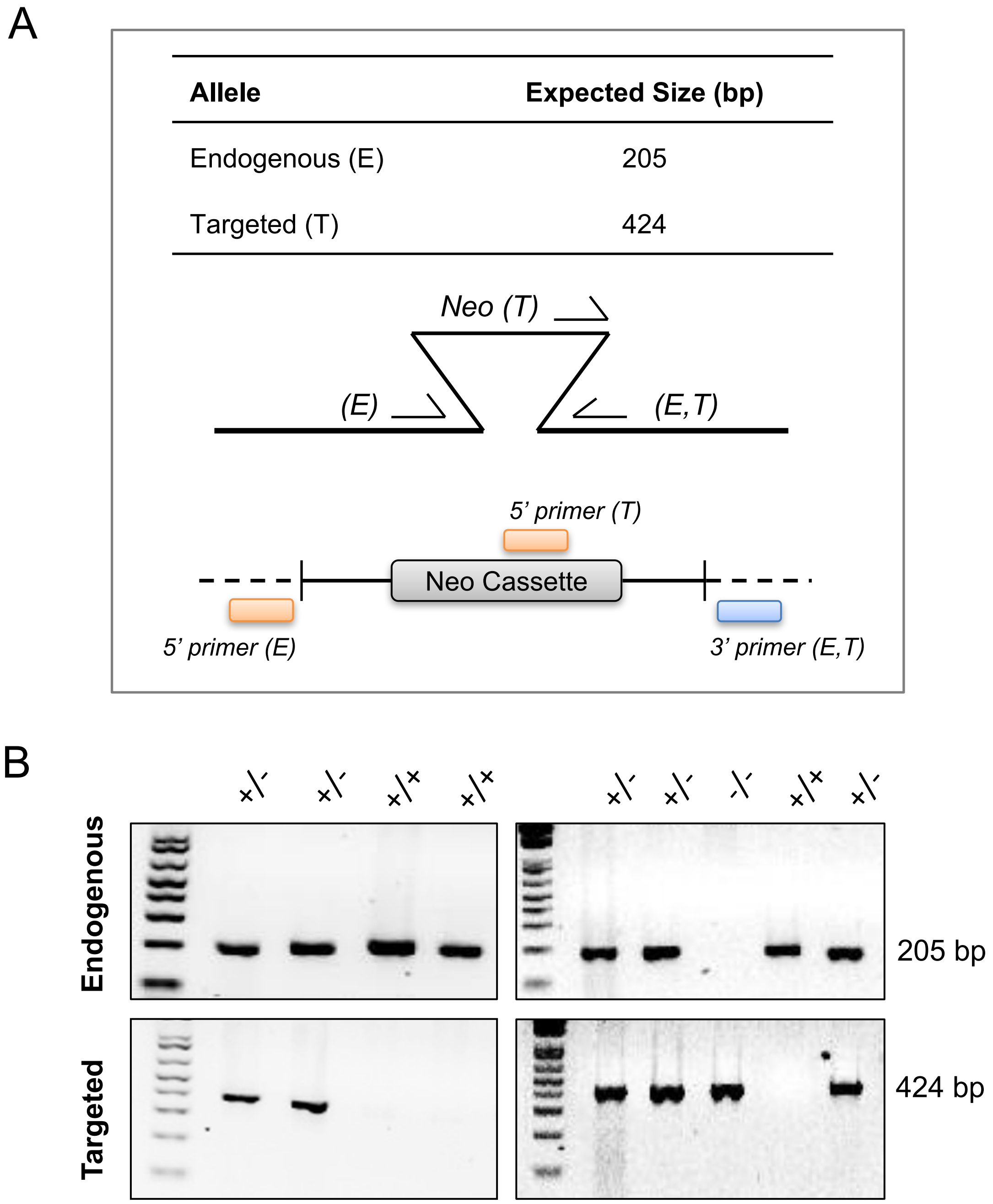

Supplementary Figure 7. (A) Scheme of PCR genotyping employed to detect both endogenous (E) and targeted (T) alleles. A first reaction is designed to detect the T allele. A second reaction only detects the E allele. Expected product sizes for each PCR reaction (E and T), in base pairs (bp) (top) and schematic diagram of the ERV1/ChemR23 knockout construct (bottom). **(B)** Gel images of PCR products of an offspring composed by 3 wild-type (+/+), 5 heterozygous ERV1/ChemR23+/− and 1 homozygous ERV1/ChemR23−/− littermates.

Supplementary Table 1. Identification, alleles, location and references of the SNPs analyzed in the study.

SNP ID.	Gene	Alleles	Chromosome	Position	References
rs1143634	IL-1β	G/A	2	112832763-112832863	Manica-Cattani MF, et al. *Mol Cell Endocrinol* 2010.<br>Carter KW et al. Hum Genet 2008.
rs1800795	IL-6	C/G	7	22726976-22727076	Yu Z et al. *Obesity* 2012.<br>Carulli L et al. Dig. Liver Dis 2009.
rs8069645	STAT3	A/G	17	42342834-42342934	Phillips CM et al. *J Nutr*. 2009.
rs7849191	JAK2	C/T	9	4988711-4988811	Pneas-Steinhardt A et al. *BMC Med Genet* 2011.
rs1800871	IL-10	A/G	1	206773239-206773339	Scarpelli D et al. Diabetes 2006.
rs8064821	SOCS3	C/A	17	78361260-78361360	Jamshidi Y et al. *Diabetologia* 2006.<br>Reid-Lombardo KM. Ann Surg 2013.
rs1878022	ERV1/ChemR23*	C/T	12	108305205-108305305	Wu X et al. J Natl Cancer Inst. 2011.<br>Alcaraz-Quiles et al. Hepatology 2017.

**The ERV1/ChemR23 gene is also known as CMKLR1.*

Supplementary Table 2: Phenotypic characteristics of ERV1/ChemR23 knockout (ERV1/ChemR23$^{-/-}$) mice compared to their wild-type (WT) littermates.

	**WT** (n= 12)	**ChemR23$^{-/-}$** (n= 12)
Body weight (g)	22.1 ± 1.2	22.3 ± 1.9
Adipose tissue/body weight	0.01 ± 0.002	0.01 ± 0.001
Liver/body weight	0.04 ± 0.005	0.05 ± 0.002
Total Cholesterol (mg/dL)	76. 6 ± 5.5	74.33 ± 5.8
HDL-c (mg/dL)	22.3 ± 3.7	23.6 ± 3.1
LDL-c (mg/dL)	39.8 ± 4.3	40.9 ± 3.6
Triglycerides (mg/dL)	72.57 ± 9.3	65.0 ± 6.9

HDL-c: high-density lipoprotein cholesterol; LDL-c: low-density lipoprotein cholesterol.
*Results are expressed as mean ± SEM.*P<0.05 versus WT.*
